# Ion Channel Targeted Mechanisms of Anti-arrhythmic Chinese Herbal Medicine Xin Su Ning

**DOI:** 10.3389/fphar.2019.00070

**Published:** 2019-02-06

**Authors:** Taiyi Wang, Weiwei Xie, Jiahui Yu, Clive Ellory, Robert Wilkins, Yan Zhu, Yu-ling Ma

**Affiliations:** ^1^Department of Physiology, Anatomy and Genetics, University of Oxford, Oxford, United Kingdom; ^2^Tianjin State Key Laboratory of Modern Chinese Medicine, Tianjin University of Traditional Chinese Medicine, Tianjin, China

**Keywords:** anti-arrhythmic drugs, premature ventricular contractions, Xin Su Ning, Chinese Herbal Medicine, electrophysiology

## Abstract

Xin Su Ning (XSN) is a China patented and certified herbal medicine used to treat premature ventricular contractions (PVCs) since 2005. A recent completed clinical trial of 861 patients showed that XSN had similar PVC inhibition rate to the class I antiarrhythmic drug mexiletine, at 65.85% for XSN and 63.10% for mexiletine. We have previously reported that XSN prolongs action potential duration (APD) and suppresses action potential amplitude (APA) of the cardiac ventricular myocytes. In this report we aim to reveal the effect of XSN on the ionic channels that govern APD and APA, which would help to explain the cellular electrophysiological mechanism of XSN. Our main findings are: (1) On ECG recorded in isolated rat, in the presence of XSN the amplitude of R wave was significantly decreased and the amplitude of T wave was increased significantly; (2) XSN blocked hNaV1.5 channel stably transfected cell line in a dose-dependent manner with an IC_50_ of 0.18 ± 0.02 g/L; and (3) XSN suppresses hERG channels in a dose-dependent manner with an IC_50_ of 0.34 ± 0.01 g/L. In conclusion, the clinical antiarrhythmic efficacy of XSN is based on its class I and Class III antiarrhythmic properties by suppression hNaV1.5 channel and hERG channels, which are directly responsible for XSN’s effect on APA suppression and APD prolongation.

## Introduction

Although much research has been carried out in the attempt to gain better understanding of cardiac arrhythmia, and in the search for effective and safe drugs to treat this common cardiac condition, the progress has been not very encouraging due to the multifactor and dynamic nature of the disease’s causes and its development (Rosen, 1988; Weiss et al., 2015). New drug development strategy of targeting ion channels or cellular electrophysiological properties have developed sophistically in the last decades, however the drugs discovered in this scheme are far from satisfactory, due to adverse reactions of the antiarrhythmic drugs (Williams, 1992; Ma et al., 2006; Frommeyer and Eckardt, 2016; O’rourke et al., 2016; Sun et al., 2016). In this report we aimed to study the antiarrhythmic mechanism of a clinically effective multi-herbal/multicomponent Chinese medicine XSN, which we hope to elicit the cellular electrophysiological property of XSN and to provide guidance for clinicians who use XSN to treat cardiac arrhythmia patients, we also hope to open up a research field to understand the antiarrhythmic actions of multicomponent medicine.

Xin Su Ning (XSN) is a multi-herbal medicine patented and certified to produce in China. It has been available in China since 2005 for treating cardiac ventricular arrhythmia, especially arrhythmias induced by cardiac ischemia and viral myocarditis (Zhai et al., 2017). XSN is comprised of 11 herbs: Coptidis Rhizoma (Huanglian, *Coptis chinensis* Franch.), Pinelliae Rhizoma [Banxia, *Pinellia ternata* (Thunb.) Makino], Poria [Fuling, *Poria cocos* (Schw.) Wolf], Aurantii Fructus Immaturus (Zhishi, *Citrus aurantium* L.), Dichroae Radix (Changshan, *Dichroa febrifuga* Lour.), Nelumbinis Plumula (Lianzixin, *Nelumbo nucifera* Gaertn.), Sophorae Flavescentis Radix (Kushen, *Sophora flavescens* Ait.), Artemisiae Annuae Herba (Qinghao, *Artemisia annua* L.), Ginseng Radix et Rhizoma (Renshen, *Panax ginseng* C. A. Mey.), Ophiopogonis Radix (Maidong, *Ophiopogon japonicus* (L. f) Ker Gawl.), and Nardostachyos Radix et Rhizoma (Gancao, *Glycyrrhiza uralensis* Fisch.).

The pre-licensing pharmacological studies in China showed that XSN significantly suppressed cardiac arrhythmia induced by the chemical reagents Matrin, CaCl_2_, Chloroform, and Isoproterenol. In cardiac ischemia-induced arrhythmia, XSN significantly delayed the onset of ventricular arrhythmia and shortened the time of arrhythmia. The pre-licensing toxicity study showed that XSN did not induce any abnormal changes to blood and urine, and the application of XSN did not cause any toxic reactions in the heart, the liver or the kidney. A recent clinical study of 861patients showed that the inhibition rate of the PVC by XSN was similar to that of mexiletine, at 65.85% for XSN and 63.10% for mexiletine (Zhai et al., 2017). An earlier clinical study reported that XSN significantly improved the overall symptoms of patients with tachycardia-associated conditions compared with propafenone (*P* < 0.05) (Yuan and Zhou, 2000). In addition, since XSN was licensed for use clinically to treat cardiac arrhythmias, there have been no adverse reactions induced by XSN being reported.

With increasing popularity of the complementary and alternative medicine among patients with PVCs, TCM (Traditional Chinese Medicine) has been more frequently used both in China and in the western countries (Zou et al., 2011; Hua et al., 2015; Wang et al., 2016). There are other antiarrhythmic Chinese medicine available in China, such as Wenxin Keli (WK) and Shen Song Yang Xin Capsule (SSYX). It was reported that WK has cardio-protective and antiarrhythmic properties with the regulatory actions on ion channels, such as I_Na_, I_Na_L, I_to_, I_CaL_, and I_f_ (Burashnikov et al., 2012; Tian et al., 2018). The other well-known antiarrhythmic TCM is SSYX. It was reported that SSYX blocks various ion channels such as I_Na_, I_CaL_, I_to_, and I_K1_ (Li et al., 2007; Zou et al., 2011).

Our previous study showed that XSN prolongs APD, a class III anti-arrhythmic characteristics in isolated cardiac myocytes (Ma et al., 2017). We report here the effect of XSN on ECG of isolated hearts, on hNav1.5 sodium channel and hERG potassium channel to illustrate a picture of the electrophysiological mechanism and to understand how XSN exerts its antiarrhythmic action clinically.

## Materials and Methods

### Medicine and Chemicals

XSN was provided by Shaanxi Momentum Pharmaceutical Co., Ltd. (Shaanxi, China) as an frozen dried powder of the extract of the 11 herbs described above (detailed preparation procedures is described in the Supplementary Material). The chemical composition of XSN was studied and has been reported recently by Guo et al. (2018), using the ultra-high-pressure liquid chromatography coupled with linear ion trap-Orbitrap tandem mass spectrometry (UHPLC-LTQ-Orbitrap), 41 components were identified as candidate bioactive components. All other chemicals were purchased from Sigma-Aldrich (Gillingham, Dorset, United Kingdom) and used as supplied unless stated.

### The Animals Used

Male Sprague-Dawley rats (280–320 g) were used for the isolated heart perfusion experiments. The rodents were housed in cages at a temperature of 22°C ± 2°C and humidity 40% ± 5%, under a 12 h light/dark cycle, and received standard diet and water *ad libitum*. All experiments were reviewed and approved by the Committee of Ethics on Animal Experiments at the TJAB (TJAB-JY-2011-002) and were carried out under the Guidelines for Animal Experiments at the Tianjin University of Traditional Chinese Medicine.

Male Wistar rats, weighing 200–250 g were used in primary cardiomyocytes isolation and were given standard laboratory chow and water *ad libitum*. All investigations conformed to the Guide for the Care and Use of Laboratory Animals published by the US National Institutes of Health (NIH Publication No. 85-23, revised 1996), the Home Office Guidance on the Operation of the Animals (Scientific Procedures) Act, 1986 (HMSO), and to institutional guidelines. Approval was granted by the University of Oxford Animal Ethics Review Committees and the Home Office (Project License numbers 30/2278 and 30/2755).

### ECG Recording

Prior the study of the effect of XSN on ion channels we carried out a group of experiment to test the effect of XSN on ECG of isolated Rat hearts. We selected a concentration that is ½ of IC50 of the APD prolongation of XSN ∼0.8 g/l in rat ventricular myocytes. The SD rats were anesthetized with isoflurane, the hearts were excised and immediately immersed in cold Krebs-Henseleit (KH) solution contained the following (120 mM NaCl, 4.7 mM KCl, 25.0 mM NaHCO_3_, 1.2 mM KH_2_PO_4_, 1.2 mM MgSO_4_, 11.1 mM glucose, 2.0 mM Na-pyruvate and 1.8 mM CaCl_2_) (Bell et al., 2011). The aorta was immediately cannulated to the Langendorff perfusion apparatus (Powerlab/8sp, ADInstruments Pty Ltd., Australia) and perfused retrogradely with KH solution (36.5 ± 1°C, pH 7.35–7.40) at a constant flow mode. KH solution was equilibrated with 95% O_2_ and 5% CO_2_ mixed gas and the perfusion speed was adjusted to 10–15 mL/min to maintain the pressure between 70 and 80 mmHg. The hearts beat spontaneously at sinus rhythm. Immediately after a stabilization period to stabilize the heart beat and state, experiment was initiated with a 15 min vehicle control period on all acceptable heart. After the perfusion of the vehicle control, the equilibrated hearts were perfused with a control KH solution or a XSN testing solution. In control group, KH solution was continuously perfused for 10 min; in XSN group, the hearts were perfused with 0.4 g/L XSN for 10 min. XSN was freshly dissolved in KH solution and filtered with 0.22 μm filter before drug application. Finally, all the hearts were perfused with KH solution for another 10 min. The following parameters were measured: R wave amplitude, T wave amplitude, QRS interval, QT interval, and RR interval.

### Primary Cardiomyocytes Isolation

The Wistar rats were anesthetized using isoflurane. Hearts were rapidly excised immediately immersed in cold Krebs-Henseleit (KH) solution described as above and perfused on a Langendorff apparatus for 15 min with Standard KH buffer at 37°C gassed with 100% O_2_, followed by changing to a low Ca^2+^ KHB solution (containing 105.1 mM NaCl, 3.0 mM KCl, 20.0 mM NaHCO_3_, 0.01 mM CaCl_2_, 1.0 mM KH_2_PO_4_, 1.2 mM MgSO_4_, 10 mM glucose, 5.0 mM Na-pyruvate, 10.0 mM taurine, and 5.0 mM mannitol, pH 7.4) for 10 min to stop the heartbeat. Then the heart was digested with recirculating low Ca^2+^ KHB solution containing 0.08 mg/mL Liberase TH (Roche-Life Science) at 37°C for 50 min. The ventricle was minced and filter into a 50 mL centrifuge tube with 4-layer gauze, centrifuged twice at 50 g for 2 min and resuspended with low Ca^2+^ KHB solution.

### Whole-Cell Patch Clamp Recording

Action potential in rat ventricular cardiomyocytes were recorded using the current clamp mode of the patch-clamp technique. cardiomyocytes were perfused at room temperature (∼22–24°C) with external solution containing in mM: NaCl, 112; NaH_2_PO_4_, 1; NaHCO_3_, 24; KCl, 5.4; MgCl_2_, 1.2; CaCl_2_, 1.8; glucose, 10; HEPES, 5; pH 7.4. Borosilicate glass electrodes (Harvard Apparatus, United Kingdom) with a tip resistance of 2∼5 MΩ were filled with the internal solution containing (in mM): KCl, 120; MgCl_2_, 2; CaCl_2_, 1; Na_2_ATP, 3; EGTA, 11; and HEPES, 10, pH 7.3. Cardiomyocytes were stimulated by applying an intracellular depolarizing stimulus with an amplitude as 2 times of action potential threshold via a digital pulse generator built in the Axopatch^TM^ 200B amplifier.

Na_V_1.5 and hERG current was recorded from stably transfected human Na_V_1.5 or hERG channel relatively using the voltage-clamp mode of patch clamp techniques (Finkel et al., 2006). The I_Na_ was activated at −120 to 20 mV sustained for 50 ms with 5 mV increments at 1 Hz. The transmembrane ion currents and potentials were recorded in the whole-cell recording mode using an Axopatch^TM^ 200B amplifier (Molecular Devices, LLC, Sunnyvale, CA, United States), and the data were analyzed using pCLAMP 10.3 software (Axon Instruments, Inc.) and Origin 9.1.

### Statistical Analysis

The data values are expressed as the mean ± standard error and analyzed using a two-tailed paired *t*-test to examine the individual apparent differences. *p* < 0.05 was used to indicate a statistically significant difference and *p* < 0.01 was used to indicate a highly significant difference.

## Results

### Effect of XSN on ECG of Isolated Rat Heart

**FIGURE 1:**
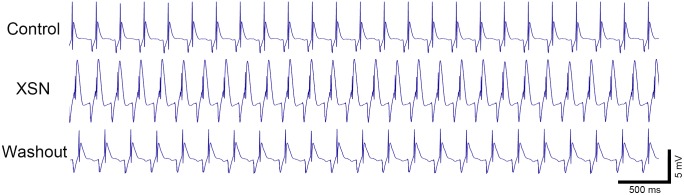
The effect of XSN on the ECG of *ex vivo* perfused rat heart. XSN at 0.4 g/L decreased the R wave amplitude, but increased T wave amplitude without changing QRS and QT interval. Heart rate (RR interval) did not show significant changes during XSN application and at 10 min washout of the medicine.

Prior the study of the effect of XSN on ion channels we carried out a group of experiment to test the effect of XSN on ECG of isolated Rat hearts with a relatively high concentration of XSN aiming to elicit the maximal effect. Compared with the control, 0.4 g/L XSN induced significant changes in the ECG morphology, including decreased amplitude of R wave and increased T wave amplitude (Figure 1 and Table 1). The QRS interval, QT interval and RR interval did not show significant alterations during the perfusion of XSN and upon the 10 min washout of XSN.

**Table 1:** Percentage change of R wave, T wave, QRS, QT, and RR interval (% of control, *n* = 5).

	R wave	T wave	QRS interval	QT interval	RR interval
XSN	54.00 ± 5.47^∗∗∗^	247.71 ± 41.12^∗^	123.72 ± 12.26	114.29 ± 10.73	91.06 ± 7.06
Washout	71.91 ± 9.93^∗^	103.31 ± 14.85	111.94 ± 7.06	116.73 ± 8.36	104.30 ± 11.86

*^∗^p < 0.05, ^∗∗∗^p < 0.001 vs. control.*

### Effects of XSN on Peak Na^+^ Current of Rat Primary Cardiomyocytes

**FIGURE 2:**
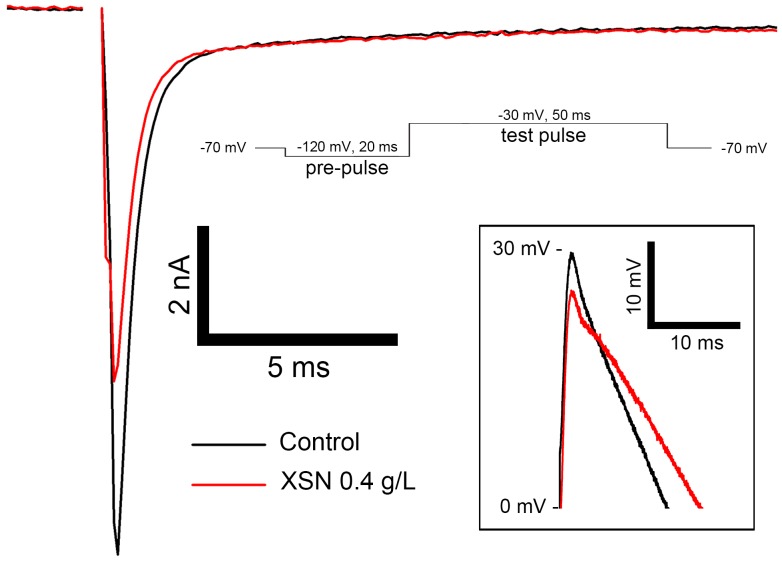
Effect of 0.4 g/L XSN on inhibition of peak Na^+^ current on primary rat ventricular myocytes (*n* = 5). The enlarged traces of the peaks of the action potentials are shown in the box on the bottom right.

The inhibitive effect of XSN on peak Na^+^ current of rat ventricular cardiomyocytes was carried in the presence of 0.4 g/L XSN. As shown in Figure 2, peak Na^+^ current was significantly suppressed by XSN which is corresponding to the suppression of the action potential amplitude.

### Effects of XSN on Human hNav1.5 Channel

**FIGURE 3:**
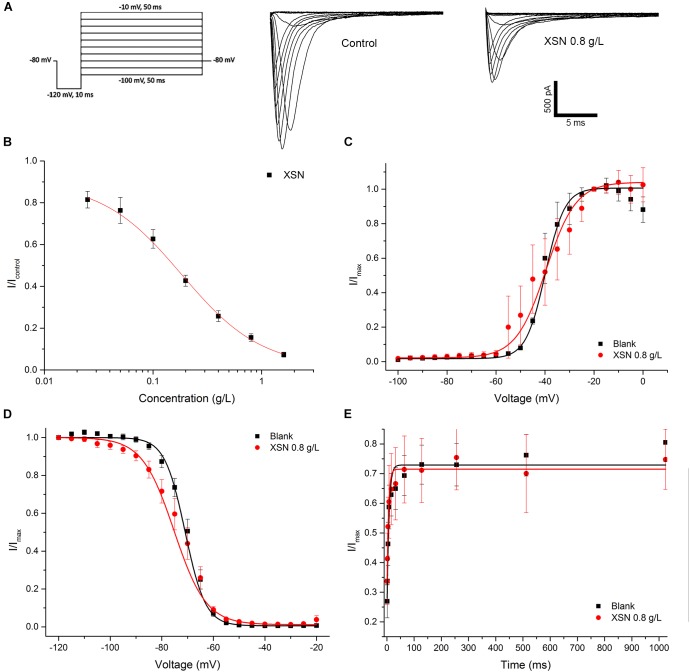
Inhibitory effect of XSN on human NaV1.5 channel. **(A)** Representative current tracing in control and in the presence of 0.8 g/L XSN. **(B)** Dose response curve of the inhibition on hNaV1.5 channel by XSN (*n* = 5). The effect of steady activation curve **(C)**, the steady inactivation curve **(D)** and the steady inactivation recovery curve **(E)** by XSN with multiple state inhibition (*n* = 5).

The inhibitory effect of XSN on hNaV1.5 channel was shown as Figure 3A. The inhibition effect of XSN on hNa_V_1.5 channel was dose-dependent with XSN concentration ranging from 0.025 to 1.6 g/L, with an IC_50_ of 0.18 ± 0.02 g/L (Figure 3B). Binding state assay showed that XSN may have the tendency to block hNaV1.5 channel at inactivation state, as shown in Figure 3C–E, V_1/2_ in steady activation curve shifted from −39.29 ± 1.41 mV (control, *n* = 8) to −39.90 ± 1.49 mV (0.8 g/L XSN, *n* = 8, *p* > 0.05 v.s. control). V_1/2_ in steady inactivation curve shifted from −70.96 ± 0.68 mV (control, *n* = 8) to −75.20 ± 0.63 mV (0.8 g/L XSN, *n* = 8, *p* < 0.001 v.s. control). tau in steady recovery curve shifted from 4.92 ± 1.24 ms (control, *n* = 6) to 3.80 ± 0.72 ms (0.8 g/L XSN, *n* = 8, *p* > 0.05 v.s. control).

**FIGURE 4:**
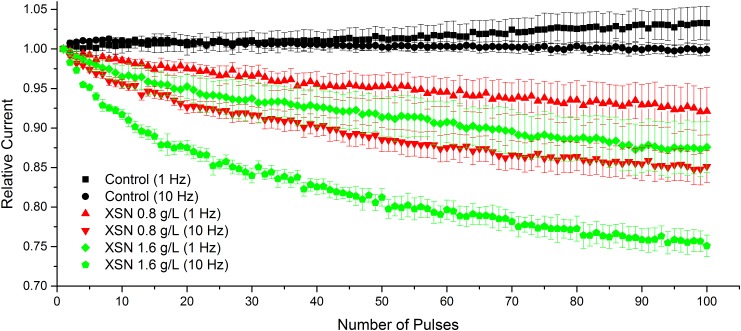
Use-dependent block of I_Na_ following acceleration from 1 to 10 Hz in control and after exposure to 0.8 and 1.6 g/L XSN. For control *n* = 12, 1 Hz *n* = 7, 10 Hz *n* = 5. Both of 0.8 and 1.6 g/L XSN in the frequency of 1 or 10 Hz showed significantly use-dependently block of Nav1.5 channel.

Compared with control condition, 0.8 g/L XSN caused significant use-dependent block at stimulation frequencies of 1 Hz (*p* < 0.001) and 10 Hz (*p* < 0.001), which was accentuated when 1.6 g/L of XSN (*p* < 0.001) was applied (Figure 4).

### Effects of XSN on Human hERG Potassium Channel

**FIGURE 5:**
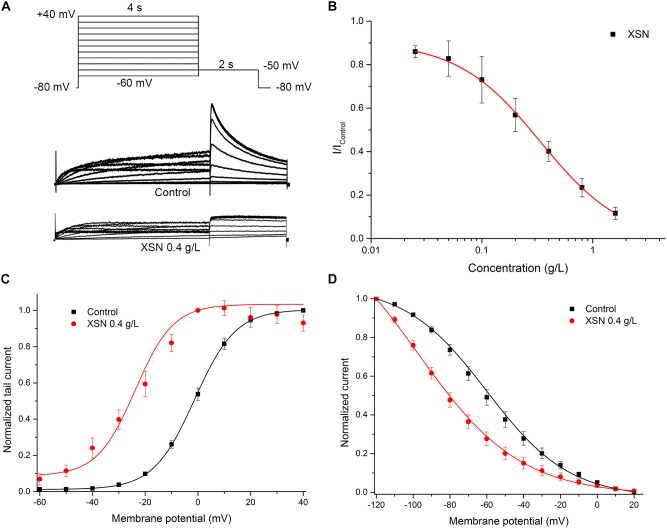
Inhibitory effect of XSN on hERG channel. **(A)** Representative current tracing in control and in the presence of 0.4 g/L XSN. **(B)** Dose response curve of the inhibition on hERG channel by XSN (*n* = 5). The effect of steady activation curve **(C)**, the steady inactivation curve **(D)** by XSN with multiple state inhibition (*n* = 8).

The effect of XSN (0.4 g/L) on the hERG channel was shown as Figure 5A. XSN dose-dependently blocked hERG channel with an IC_50_ = 0.34 ± 0.01 g/L (Figure 5B). Steady-state inhibition assay showed that XSN may have the tendency to block hERG channel at both open and inactivation state, as shown in Figure 5C,D. V_1/2_ in steady-state activation curve shifted from −1.27 ± 0.28 mV (control, *n* = 8) to −23.79 ± 2.31 mV (0.4 g/L XSN, *n* = 8, *p* < 0.001 v.s. control). V_1/2_ in steady inactivation curve shifted from −59.87 ± 0.81 mV (control, *n* = 8) to −97.56 ± 2.25 mV (0.4 g/L XSN, *n* = 8, *p* < 0.001 v.s. control).

## Discussion

XSN is a clinically proven antiarrhythmic medicine in China (Yuan and Zhou, 2000). It has been widely used in China for more than 10 years, without sever adverse reactions being reported. It is applicable for moderating premature ventricular contraction (PVC) caused by coronary heart disease and viral myocarditis (Lin et al., 2011). In 2017, the clinical trial of 861 patients was reported in Lancet. The results showed that the mean reduction of PVC by XSN (4604.67 ± 6990.07) was close to Mexiletine (4502.86 ± 5771.70) and was significantly higher than placebo group (1512.47 ± 8311.14). In agreement with the more than 10 years clinical use, there had no pro-arrhythmic phenomenon of XSN being seen in this clinical study. Furthermore, XSN has been reported to treat paroxysmal atrial fibrillation and viral myocarditis (Wang and Lu, 2008; Li and Zhang, 2015). Our previous cellular electrophysiological study showed that XSN displays the property of Class III antiarrhythmic drugs by prolong APD of the cardiac myocytes. In this study, we revealed that XSN blocks hNaV1.5 sodium channel and hERG potassium channel, which has placed XSN as a class I and III antiarrhythmic medicine as the widely used antiarrhythmic drug amiodarone.

Our results presented here furthered our understanding of the clinical anti-arrhythmic action of XSN: (1) in the *ex vivo* ECG study on isolated wistar rat heart, the R wave and T wave of the ECG showed significant morphological changes after the application of XSN (Figure 1). It is well known, the QRS complex, especially the R wave amplitude, represents ventricular depolarization caused by transiently activation of sodium channels. The suppression of R wave suggests the inhibition of fast-voltage-gated sodium channel, which is the anti-arrhythmic mechanism of the Class I anti-arrhythmic agents. In fact, the clinical dose of XSN in human blood serum was much lower than 0.4 g/L, even though, at this concentration, XSN did not induce Torsade de Pointes (TdP) or other kind of arrhythmia in the isolated rat hearts, which may well be the beneficial outcome of the multicomponent action of XSN.

The cellular electrophysiological assay showed that XSN prolonged APD_90_ on rat cardiomyocytes (Ma et al., 2017) and reduced the APA as shown in Figure 2, which indicated that XSN may block sodium and potassium channels. These indications were supported by our results shown in Figure 2, 3.

The inhibitory effect of XSN on human Nav1.5 channel shown in Figure 3 displays a dose dependent manner at concentrations ranging from 0.025 to 1.6 g/L XSN, and that the human Na_V_1.5 channel was blocked at inactivation state as shown in Figure 3. As peak sodium and late sodium current are produced by Na_V_1.5 channel (Maltsev et al., 2009), the inactivation blockage of Na_V_1.5 suggests the possibility of a post-repolarization refractoriness period, which provide mechanistic explanation for XSN’s inhibition of the premature hearts beat in patients. Additionally, 0.8 g/L XSN caused significant use-dependent block at both frequencies tested (Figure 4): 1 Hz (*p* < 0.001) and 10 Hz (*p* < 0.001), which helps to explain the clinical therapeutic effect of XSN on tachycardia (Hondeghem and Katzung, 1977; Campbell, 1983; Wang et al., 2015; Potet et al., 2016).

XSN blocks hERG potassium channel which would prolong APD and increase the effective refractory period of the heart which in turns inhibits PVC.

PVCs are a relatively common occurrence caused by irritated ectopic foci in the cardiac ventricle (Adams et al., 2012), which are responsible for considerable morbidity and mortality (Takemoto et al., 2005). It may cause hemodynamic deterioration and reversible left ventricular dysfunction and can act as electrophysiological markers to other cardiac diseases such as cardiomyopathy and ischemic heart disease (Zaborska et al., 2006; Kanei et al., 2008). It has been demonstrated that the quantitative additional mortality risk on exercise test presented by frequent PVCs is similar to ischemia (Iacovino, 2001). Clinical presentation of PVCs may be quite variable, ranging from an incidental finding on electrocardiogram (ECG) to congestive heart failure (Morganroth, 1984; Santoro et al., 2014). For patients with symptoms that can be attributed to PVCs. the drug therapy of beta blockers or class I or III antiarrhythmic agents is effective and recommended (Zipes et al., 1984; Capucci et al., 1991; Zou et al., 2011; Zhong et al., 2014).

XSN, as a multicomponent medicine, inhibit PVC, ischemia induced cardiac arrhythmia and virus myocarditis, which show an abroad spectrum pharmacological actions. As we know that arrhythmias more often is induced by other underlying cardiac conditions, therefore it is a multifactor disease which would need multicomponent medicine to treat. XSN has displayed class I and III antiarrhythmic properties, but did not induce arrhythmia at very high concentration, we are aiming to further our study to discover how the multicomponent in XSN coordinated to achieve the therapeutic efficacy without causing adverse reactions.

## Conclusion

Our results presented here have proved further that multi-herbal antiarrhythmic medicine XSN displays clear cellular electrophysiological mechanism that support its clinical efficacy in patients, and the results also assemble the properties processed by antiarrhythmic chemical drugs although the data present the overall effect of a multicomponent medicine. We therefore conclude that: (1) XSN is a class I and III antiarrhythmic multicomponent medicine; (2) The dose-dependent and completely reversible effect of XSN indicate a low toxicity property of XSN; (3) The discoveries that XSN blocks hNav1.5 and hERG backed by the PAP suppression and prolongation of APD are the cellular electrophysiological mechanism that support the clinical therapeutic effect of XSN in treating PVC and tachyarrhythmia in patients.

## Study Limitations

Although our results have clearly placed XSN into the groups of class I and III antiarrhythmic drugs, the effect of XSN in animal arrhythmic models is lacking in this report. Since there is strong evidence that XSN is a clinically effective antiarrhythmic medicine in treating PVC and ischemia induced cardiac arrhythmia, it would add valuable laboratory research data if we study the effect of XSN in animal ischemia and arrhythmia models. We are aiming in our future report to demonstrate the *in vivo* antiarrhythmic effect of XSN.

## Author Contributions

Y-lM conceived and designed the experiments. TW, WX, and JY performed the experiments. TW and Y-lM analyzed the data. YZ and Y-lM contributed reagents, materials, and analysis tools. TW and Y-lM wrote the paper. CE and RW discussed the experimental design.

## Conflict of Interest Statement

The authors declare that the research was conducted in the absence of any commercial or financial relationships that could be construed as a potential conflict of interest.
