# Corrigendum: Ion Channel Targeted Mechanisms of Anti-arrhythmic Chinese Herbal Medicine Xin Su Ning

**DOI:** 10.3389/fphar.2019.00493

**Published:** 2019-05-09

**Authors:** Taiyi Wang, Weiwei Xie, Jiahui Yu, Clive Ellory, Robert Wilkins, Yan Zhu, Yu-ling Ma

**Affiliations:** ^1^Department of Physiology, Anatomy and Genetics, University of Oxford, Oxford, United Kingdom; ^2^Tianjin State Key Laboratory of Modern Chinese Medicine, Tianjin University of Traditional Chinese Medicine, Tianjin, China

**Keywords:** anti-arrhythmic drugs, premature ventricular contractions, Xin Su Ning, Chinese Herbal Medicine, electrophysiology

In the original article, we incorrectly used the university internal grant code which should be replaced by the Chinese Medicine Research Fund in University of Oxford. The authors apologize for this error and state that this does not change the scientific conclusions of the article in any way. The original article has been updated.

The funding statement should read:

This work was supported by the Chinese Medicine Research Fund in University of Oxford. The grant was sponsored by Shaanxi Momentum Pharmaceutical Co., Ltd.

Shaanxi Momentum Pharmaceutical Co., Ltd. had no involvement in the study design, data collection and analysis, decision to publish, or preparation of the manuscript. All authors declare no conflict of interest.

