# Supplementary material for: Ion Channel Targeted Mechanisms of Anti-arrhythmic Chinese Herbal Medicine Xin Su Ning
Source: Front Pharmacol. 2019 Feb 6;10:70. doi: 10.3389/fphar.2019.00070 (PMC6372541; doi:10.3389/fphar.2019.00070)
Supplement: Supplementary file 1 [file Data_Sheet_1.PDF]

Supplementary material

The detailed information of Xin Su Ning

1. Herbal ingredients of XSN

Table S1 The names and weight portion of 11 herbs in XSN

Herb name	Latin name	Origin plants	Weight
Huanglian	Coptidis Rhizoma	*Coptis chinensis* Franch.	334 g
Banxia	Pinelliae Rhizoma	*Pinellia ternata* (Thunb.) Makino	250 g
Fuling	Poria	*Poria cocos* (Schw.) Wolf	250 g
Zhishi	Aurantii Fructus Immaturus	*Citrus aurantium* L.	167 g
Changshan	Dichroae Radix	*Dichroa febrifuga* Lour.	250 g
Lianzixin	Nelumbinis Plumula	*Nelumbo nucifera* Gaertn.	42 g
Kushen	Sophorae Flavescentis Radix	*Sophora flavescens* Ait.	250 g
Qinghao	Artemisiae Annuae Herba	*Artemisia annua* L.	250 g
Renshen	Ginseng Radix et Rhizoma	*Panax ginseng* C. A. Mey.	167 g
Maidong	Ophiopogonis Radix	*Ophiopogon japonicus* (L. f) Ker Gawl.	250 g
Gancao	Nardostachyos Radix et Rhizoma	*Glycyrrhiza uralensis* Fisch.	167 g

2. Procedure of preparing XSN (Wang and Mao, 2018)

(1) The foreign matters and non medicinal parts are picked out from Artemisiae Annuae Herba, Sophorae Flavescentis Radix, Glycyrrhizae Radix Et Rhizoma, Poria, Coptidis Rhizoma, Aurantii Fructus Immaturus, Dichroae Radix, Ginseng Radix Et Rhizoma, Pinelliae Rhizoma, Ophiopogonis Radix, and Nelumbinis Plumula, respectively, and then set aside until use; The foreign matters and non medicinal parts are picked out from Artemisiae Annuae Herba, Sophorae Flavescentis Radix, Glycyrrhizae Radix Et Rhizoma, Poria, Coptidis Rhizoma, Aurantii Fructus Immaturus, Dichroae Radix, Ginseng Radix Et Rhizoma, Pinelliae Rhizoma, respectively, and then rinsed (sprayed) or moistened (slightly moistened) with flowing water and cut into slices or segments; Ophiopogonis Radix is moistened with a flowing water then crashed as flate and set aside until use. Dried at 70~80° C.

(2) Coptidis Rhizoma, Aurantii Fructus Immaturus, Dichroae Radix, Nelumbinis Plumula and Sophorae Flavescentis Radix are washed with 20 kg tap water for two times, and rinsed with 10 kg purified water for two times, then extracted by reflux with 60% ethanol for two times, add 8.4 L for the first time and 6.3 L for the second time, 1.5 h for each time. Combined extract, filtrated, and the filtrate is concentrated in vacuum at 0.04 Kpa and recovered ethanol at 70° C. Then 6.3 L injection water is added, heated at 50° C.~60° C., stirred to make it fully dissolved， filtrated while hot, stand for overnight, the supernatant is set aside until use;

(3) Group B, Ginseng Radix Et Rhizoma, Pinelliae Rhizoma and Poria are washed with 13 kg tap water for two times, and rinsed with 6.5 kg purified water for two times, then

extracted by reflux with 70% ethanol for two times, add 5.3 L for the first time and 4 L for the second time, 2 h for each time. Combined extract, filtrated, the filtrate and residue are set aside until use, respectively;

(4) Group C, Ophiopogonis Radix, Artemisiae Annuae Herba and Glycyrrhizae Radix Et Rhizoma are washed with 13 kg tap water for two times, and rinsed with 6.5 kg purified water for two times, then combined with the residue in group B, decocted with injection water for two times, add 6.7 L for the first time and 5.3 L for the second time, 1 h for each time. Combined the filtrates for the second time and discarded the residue, the filtrate is concentrated to obtain liquid extract with the relative density ranging from 1.05 to 1.06 (80° C.) and then 95% ethanol is added to the alcohol content reaching 70%, stirred well and stand for 24 h. The supernatant is combined with the filtrate in group B, and recovered ethanol at 70° C. Then add 4 L injection water, heated at 50° C ~60° C., stirred to make it fully dissolved, filtrated while hot, stand for overnight, the supernatant is set aside until use;

(5) Combined the supernatant of group A and C, then concentrated to obtain liquid extract with the relative density of 1.10 (60° C.) by rotating film evaporator and set aside until use;

(6) Fixed the liquid extract with the relative density of 1.10 (60° C.) in pallet, freeze-dried for 20 h below -40°C. with the vacuum below 15 Pa and the heating temperature from -20°C. to 80°C. Taken out and collected the freeze-dried solids, crushed, mixed, then 590 g lyophilized powder is obtained.

Wang, B., and Mao, P. (2018). "Use of traditional chinese medicine composition in preparation of potassium ion channel modulator medicine". Google Patents.
